# PlGF knockdown attenuates hypoxia-induced stimulation of cell proliferation and glycolysis of lung adenocarcinoma through inhibiting Wnt/β-catenin pathway

**DOI:** 10.1186/s12935-020-01714-w

**Published:** 2021-01-06

**Authors:** Wei Zhang, Yanwei Zhang, Wensheng Zhou, Fangfei Qian, Minjuan Hu, Ya Chen, Jun Lu, Yuqing Lou, Baohui Han

**Affiliations:** Department of Pulmonary Medicine, Shanghai Chest Hospital, Shanghai Jiao Tong University, Shanghai, 200030 People’s Republic of China

**Keywords:** Hypoxia, Cell proliferation, Glycolysis, β-catenin, C-myc

## Abstract

**Background:**

Angiogenic placental growth factor (PlGF) plays a role in hypoxia-induced angiogenesis. Here, we aimed to investigate the biological roles of PlGF in cell proliferation and glycolysis of lung adenocarcinoma (LUAD) and the underlying molecular mechanisms.

**Methods:**

PlGF was knocked down in H358 and H1975 cells by lentiviruses, which were then cultured under hypoxia (90% N_2_, 5%CO_2_ and 5%O_2_) for 24 h. PlGF was overexpressed in PC9 cells treated with XAV939, inhibitor of Wnt/β-catenin signaling pathway. PlGF-silencing H1975 cells were implanted into mice, and tumor xenografts were harvested and analyzed.

**Results:**

Hypoxia treatment led to up-regulation of PlGF, C-myc, lactate dehydrogenase A (LDHA), and β-catenin, promotion of cell proliferation and glycolysis in H358 and H1975 cells, which were obviously reversed by knocking down PlGF. In tumors, PlGF knockdown significantly prohibited cell proliferation and glycolysis, and decreased expression of C-myc, LDHA, and β-catenin. PlGF overexpression markedly strengthened cell proliferation, which was inhibited by β-catenin knockdown. Consistently, XAV939, inhibitor of Wnt/β-catenin pathway, also inhibited PlGF-induced cell proliferation, glycolysis, and β-catenin expression in PC9 cells.

**Conclusion:**

PlGF knockdown inhibited the stimulatory effect of hypoxia on cell proliferation and glycolysis of LUAD through deactivating Wnt/β-catenin pathway.

## Background

Hypoxia is a widely-accepted characteristic of cancer development with a significant mutagenic potential, which refers to a mismatch between oxygen demand and supply in tissues resulted from rapidly proliferating cancer cells [1, 2]. Hypoxia is critical for cancer progression, invasion and treatment resistance by regulating vascularization, metabolism, cell survival and apoptosis [3, 4]. Hypoxia-inducible factor-1 (HIF-1) is the critical mediator of the hypoxic condition [5]. Studies have revealed that HIF-1 participates in regulating expression of a great number of oxygen-dependent proteins involved in angiogenesis and cell metabolism, such as vascular endothelial growth factor (VEGF), and placental growth factor (PlGF) [6–8].

To adapt to hypoxic microenvironment, oxidative phosphorylation is switched to glycolysis in tumor cells, which not only provide cellular energy, but also foster macromolecular biosynthesis by the production of metabolic intermediates, thereby promoting tumor growth, and facilitating immune escape [9, 10]. Increased glycolysis is one of the metabolic characteristics known as the Warburg effect, which is the object of debate in the pathogenesis of tumors recent years [11, 12]. Oxygen consumption rates (OCR) is an indicator of oxygen consumption by the cells, while extracellular acidification rate (ECAR) is used as an marker of glycolytic respiration by evaluating the production of lactic acid in the medium outside the cells [13]. Glycolysis inhibition has been increasingly recognized as a novel strategy to combat cancer [14]. Previous studies have made considerable efforts to unravel the physiological and molecular mechanisms behind glycolysis enhancement in tumor and have unveiled a few transcription factors as central regulators, such as HIF-1 and C-myc [15, 16]. Oncogene MYC encodes C-myc of the Myc family, which is a transcription factor involved in cell proliferation, apoptosis and metabolism, contributing to tumorigenesis [17]. C-myc modulates the genes involved in the biogenesis of ribosomes and mitochondria, glucose and glutamine metabolism [18]. C-myc is a key regulator of glycolysis and can transcriptionally elevate expression of lactate dehydrogenase A (LDHA), hexokinase 2 and pyruvate kinase isoform 2, which are important players in glycolysis [19].

PlGF is a proangiogenic protein belonging to the VEGF family, which is implicated in vasculogenesis and angiogenesis [20]. Increasing studies have proved that PlGF is involved in the control of metastasis and vascularization of lung cancer [21, 22]. Previously, we have found that PlGF promotes invasion of non-small cell lung cancer via matrix metalloproteinase-9 [23]. However, the associations between PIGF and promotion of tumor growth and glycolysis by hypoxia remain poorly understood. Here, we attempted to investigate the biological action of PlGF in lung adenocarcinoma (LUAD) cells under hypoxic condition and decipher the underlying molecular mechanisms. In our study, up-regulation of PlGF was firstly observed in cancer tissue. We knocked down and overexpressed PlGF in LUAD cells by using lentivirus so as to explore its effects on cell proliferation and glycolysis in response to hypoxia. Tumor xenografts in nude mice were adopted to verify its biological role as well. This study would deepen our understanding of the involvement of PlGF in the pathogenesis of LUAD.

## Materials and methods

### Bioinformatics analysis

Gene expression data of 515 primary LUAD samples and 59 paired normal lung samples were downloaded from The Cancer Genome Atlas (TCGA) (https://gdc-portal.nci.nih.gov/) as our previous described [24]. Expression level of PlGF was compared by LUAD and normal samples by use of 3.34.7 limma package [25] of R language. Overall survival time of patients with high and low PlGF expression was analyzed and compared by using Kaplan–Meier survival analysis and log-rank t test. Pathway enrichment analysis was carried out using gene set enrichment analysis software [26].

### Immunohistochemistry

Immunohistochemistry (IHC) was performed on purchased tissue microarrays at room temperature as described previously [27]. Briefly, paraffin-embedded tissue microarrays were blocked in 1% BSA (A8010, Solarbio, China) for 1 h. Subsequently, the tissue slices were incubated in specific primary antibodies against PlGF (ab196666, Abcam, Cambridge, UK) and β-catenin (ab16051, Abcam, Cambridge, UK) for 1 h, followed by incubation in HRP-labeled secondary antibodies (D-3004, Changdao, China) for 30 min. The tissue slices were stained with DAB (FL-6001, Changdao, China) for 5 min, and stained with hematoxylin (714094, BASO, Wuhan, China) for 3 min. Finally, the slices were examined on a microscope (ECLIPSE Ni, NIKON, Japan).

### Cell culture and hypoxic treatment

Cell culture were performed as our previous studies [28–30]. RPMI-1640 medium (Hyclone, Logan, UT, US) was used to culture lung cancer cells (A549, H1975, H1650, H358, PC9) and human bronchial epithelial cell (16HBE). H1975 and H358 cells were cultured in a hypoxic chamber mixed with anaerobic gas (90% N_2_, 5% CO_2_) and 5% O_2_ (hypoxia treatment) or in an incubator of 5% CO_2_ and 95% air (normoxia treatment) for 24 h.

### Quantitative real-time PCR (qRT-PCR)

According to the manufacturers’ instructions, briefly, RevertAid First Strand cDNA Synthesis Kit (Fermentas, USA) was applied to transcribe the RNA samples into cDNA through reverse transcription. SYBR Green qPCR Master Mixes (Thermo Fisher, USA) was applied to amplify the cDNA product. The primer sequences used for PlGF, HIF-1α and GAPDH were listed in Table 1.

**Table 1:** The primer sequences used for qRT-PCR

Primer	Sequences
PlGF-primer F	5′-CATGTTCAGCCCATCCTGTG-3′
PlGF-primer R	5′-ACCTTTCCGGCTTCATCTTC-3′
HIF-1α-primer F	5′-AGAGTTACCTGCCCTGTCCC-3′
HIF-1α-primer R	5′-GCCAAAACCGTCCCGAAG-3′
GADPH-primer F	5′-AATCCCATCACCATCTTC-3′
GADPH-primer R	5′-AGGCTGTTGTCATACTTC-3′
β-catenin-primer F	5′-GCCACAAGATTACAAGAAACGG-3′
β-catenin-primer R	5′-ATCCACCAGAGTGAAAAGAACG-3′

### Western blot analysis

The lysates were separated by electrophoresis and transferred onto a PVDF membrane. The membrane were then incubated with primary antibodies (1:1000) and second antibodies sequentially. The following antibodies were used, including Anti-PlGF (ab196666), anti-HIF-1α (ab51608), anti-C-myc (ab32072), anti-LDHA (ab125683), anti-Survivin (ab76424), anti-β-catenin (ab32572), anti-H3 (ab1791) from Abcam (Cambridge, UK), and anti-GAPDH (#5174) from Cell Signaling Technology (Beverly, MA, USA).

### Plasmid construction

PlGF and β-catenin interference sequences (shown in Table 2) were cloned into the pLKO.1-puro plasmid to knockdown PlGF and β-catenin. The primers enclosing the cutting sites of EcoR I and BamH I were used to synthesize the coding sequence of PlGF (X54936.1) which was merge into pLVX-Puro to promote PlGF overexpression:

**Table 2:** PlGF and β-catenin interference sequences

Name	Sequences
PlGF site 1 (583–601)	GGCGATGAGAATCTGCACT
PlGF site 2 (629–647)	CCATGCAGCTCCTAAAGAT
PlGF site 3 (636–654)	GCTCCTAAAGATCCGTTCT
β-catenin site 1 (1127–1145)	GCTTATGGCAACCAAGAAA
β-catenin site 2 (1292–1310)	GCTGGTGGAATGCAAGCTT
β-catenin site 3 (2017–2035)	GCTGCTTTATTCTCCCATT

PlGF-F: 5′-CGGAATTCATGCCGGTCATGAGGCTG-3′ (EcoR I)

PlGF-R: 5′-CGGGATCCTTACCTCCGGGGAACAGC-3′ (BamH I).

### Cell transfection

An aliquot of 2 ml of suspension (1 × 10^6^ cells/ml) was inoculated into 6-well plates for overnight culture. When 60–70% confluence was reached, lung cancer cells were transfected with shPlGF-1, shPlGF-2, shPlGF-3 (MOI = 5, 5 µl) and shNC (MOI = 5, 5 µl), or oePlGF (MOI = 5, 5 µl)and empty plasmids (vector, MOI = 5, 5 µl), or shβ-catenin-1, shβ-catenin-2, shβ-catenin-3 (MOI = 5, 5 µl) and shNC (MOI = 5, 5 µl) using Lipofectamine 2000 reagent (Invitrogen, CA, USA). The cells incubated with medium served as a control.

### Cell counting kit-8 assay

Cell counting kit-8 (CCK-8) assay was applied using a Cell Proliferation and Cytotoxicity Assay Kit (SAB, USA). Briefly, 100 ml cell suspension (2 × 10^3^) of lung cancer cells (H1975, H358 or PC9) was added to each well of a 96-well plate, followed by different cell treatments. Eventually, 10 µl of CCK-8 solution was added to each well and optimal density (OD) at 450 nm was measured.

### Cell mito stress test assay and Glycolysis stress test assay

Mitochondrial respiration experiments were conducted using the Mito Stress Test Kit and Glycolysis Stress Test Kit (Seahorse Bioscience, Billerica, MA, USA). OCR and ECAR were measured using a Seahorse the XF24 analyzer (Seahorse Bioscience, Billerica, MA, USA).

Cell mito stress test assay was performed as previously described [31]. Cells were seeded into 96-well cartridges and pre-equilibrated with the assay medium for 1 h. OCR was assayed under basal conditions followed by sequentially loading pre-warmed oligomycin, FCCP, rotenone & antimycin A into the sensor cartridge. ATP coupler oligomycin allows for measurement of oxygen consumption for ATP synthesis. Uncoupling FCCP reagent is used to measure maximal OCR level for evaluation of the spare respiratory capacity. Rotenone and antimycin A arrests mitochondrial respiration by prohibiting mitochondrial complexes I and II. Final concentrations of these reagents have been demonstrated in a previous study by Tan et al. [13].

Glycolysis stress test assay was carried out following the vendor instructions (Seahorse Bioscience, Billerica, MA, USA). Briefly, cells were calibrated by the assay medium. Glucose, oligomycin and 2-deoxyglucose (2-DG) were then sequentially added in the assay medium. ECAR was detected under basal conditions and after separate treatments of glucose, oligomycin, and 2-DG. Glucose addition promotes glycolysis, oligomycin treatment suppresses oxidative phosphorylation and permits analysis of maximal cellular glycolytic capacity, and 2-DG treatment is used to inhibit glycolysis.

### Stable cell lines and xenograft study

H1975 cells (6 × 10^6^) infected with shPlGF-2 or shNC were injected into male nude mice (4–5 week old, Shanghai Laboratory Animal Company, Shanghai, China) subcutaneously. After measurement of tumor volume every 3 days, mice were sacrificed at 33 days. Tumor xenografts were collected, weighed and analyzed, followed by immunofluorescence (IF) microscopy for detection of anti-Ki-67 (ab23345, Abcam and hematoxylin–eosin (HE) staining. All animal experiments were performed following the ethics guidelines, and were approved by the ethical committee.

### IF microscopy

Briefly, tissue sections of tumor xenografts harvested from mice were incubated with anti-Ki-67 antibody (Abcam; 1:1000 dilution), Goat anti-Rabbit IgG (H + L) antibody (Beyotime Biotechnology; 1:500 dilution) before nuclei staining with DAPI (C1002, Beyotime Biotechnology; 1:500 dilution). A Laser scanning confocal microscope (Leica Microsystems Inc., USA) was used to observe the stained cells.

### HE staining

Briefly, after embedding and fixing the tissue sections (4–7 µm thickness) were immersed in xylene (Shanghai Sinopharm) and ethanol sequentially. Staining in hematoxylin solution lasted for 5 min and staining in eosin solution lasted for 1–2 min followed by alcohol dehydration. Eventually, a NIKON microscope (ECLIPSE Ni) and MS image analysis system (DS-Ri2, NIKON, Japan) were used to observe and analyze the tissue sections.

### Statistical analysis

Data was present as mean ± SD. Statistical analysis was performed using GraphPad Prism software (version 7.0, USA), and each experiment was repeated at least three times. One-way analysis of variance (ANOVA) was applied for comparing mean values between different groups. *P *< 0.05 suggested significance.

## Results

### PlGF was up-regulated in primary tumor tissues and in lung cancer cells

**Fig. 1:**
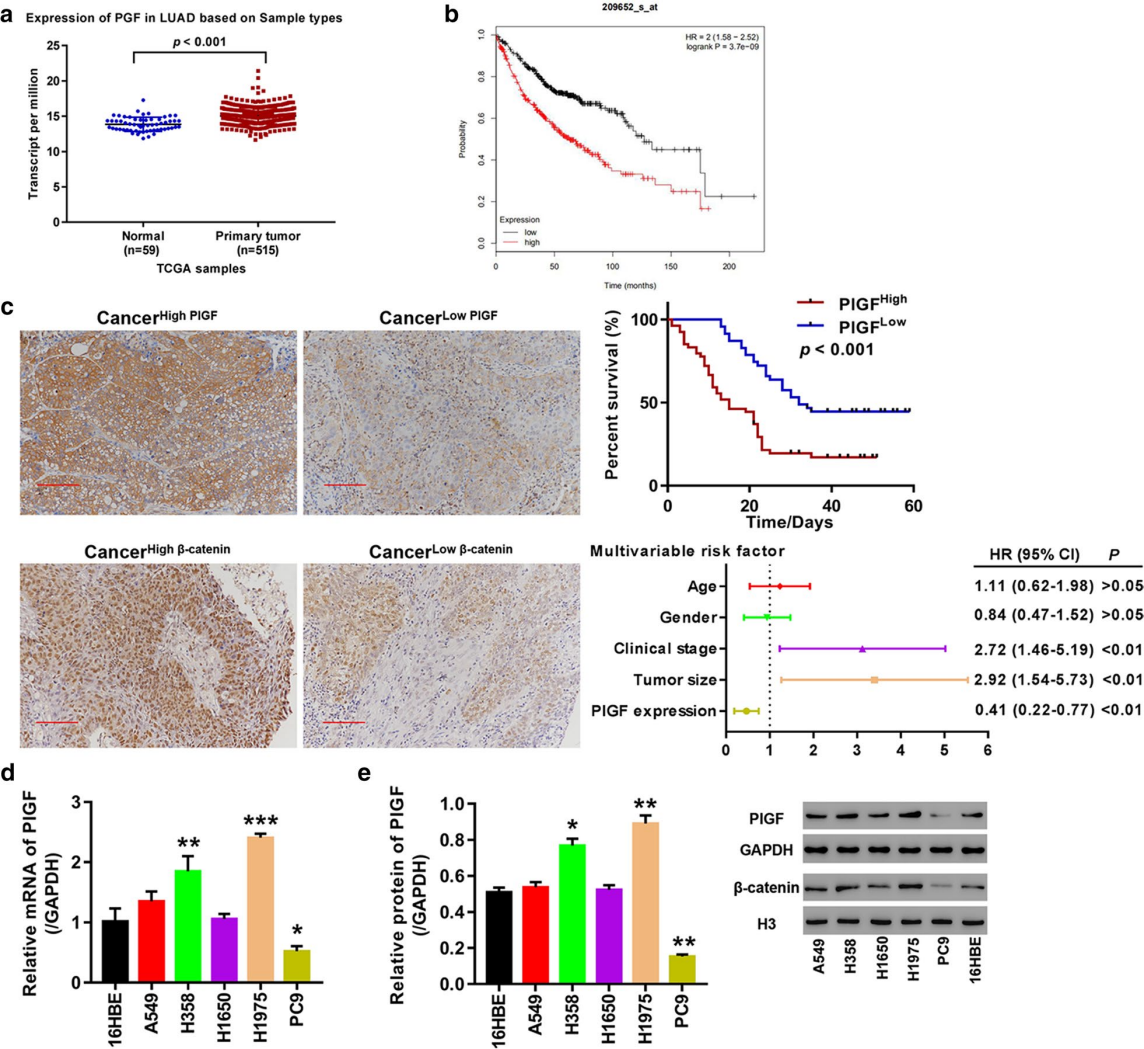
PlGF was highly expressed in lung adenocarcinoma tissue and cells. **a** Comparison of PlGF expression in normal and tumor tissues based on TCGA data; **b** Survival analysis of the patients with high and low PlGF expression; **c** Analysis of PlGF expression in cancer tissue microarrays. Left panel shows representative IHC images of PlGF and β-catenin staining in cancer and paracancer tissue microarrays (200 × magnification); upper panel shows Kaplan–Meier survival curves of the cancer tissue samples with high and low PlGF expression; lower panel displays results of multi-variable cox regression analysis; **d** PlGF mRNA levels in A549, H1975, H1650, H358, PC9and 16HBE cells; **e** PlGF protein expression in A549, H1975, H1650, H358, PC9 and 16HBE cells. *p < 0.05, **p < 0.01, ***p < 0.001 *vs.*16HBE cells

Using gene expression data of 515 primary LUAD samples and 59 paired normal lung samples downloaded from TCGA database, we found that PlGF expression was remarkably higher in LUAD tissues relative to the paired normal lung tissue (Fig. 1a). The patients with high PlGF expression had significantly poor prognosis compared to the patients with low PlGF expression (HR = 2(1.58–2.52), log-rank P = 3.7e^−09^, Fig. 1b). Representative IHC photographs of positive expression of PlGF and β-catenin in LUAD tissues microarrays showed elevated expression of β-catenin in the cancer tissues with high PlGF expression, but was decreased in cancer tissues with low PlGF expression (Fig. 1c). The patients with low PlGF expression had significant better survival compared to the patients with high PlGF expression (*p *< 0.001, Fig. 1c). Clinical stage (HR (95% CI) 2.72 (1.46–5.19); *p *< 0.01), tumor size (HR (95% CI) 2.92 (1.54–5.73); *p *< 0.01) and PlGF expression (HR (95% CI) 0.41 (0.22–0.77); *p *< 0.01) were identified to be independent risk factors of prognosis (Fig. 1c). Additionally, PlGF- and β-catenin-positive expression was obviously weaker in para-cancer tissues. We further detected mRNA and protein level of PlGF in 5 lung cancer cell lines (A549, H1975, H1650, H358, and PC9) and human bronchial epithelial cells (16HBE) by qRT-PCR and Western blot. As seen in Fig. 1d, e, the two approaches achieved consistent results that significant elevations of PlGF mRNA and protein were found in H358 cells (*p *< 0.01; *p *< 0.05) and H1975 cells (*p *< 0.001; *p *< 0.01), while significant reductions of PlGF mRNA and protein were found in PC9 cells (*p *< 0.05; *p *< 0.01), when compared with 16HBE cells. Therefore, H358, H1975 and PC9 cells were selected to be used in further experiments.

### Knockdown and overexpression of PlGF in lung cancer cells

**Fig. 2:**
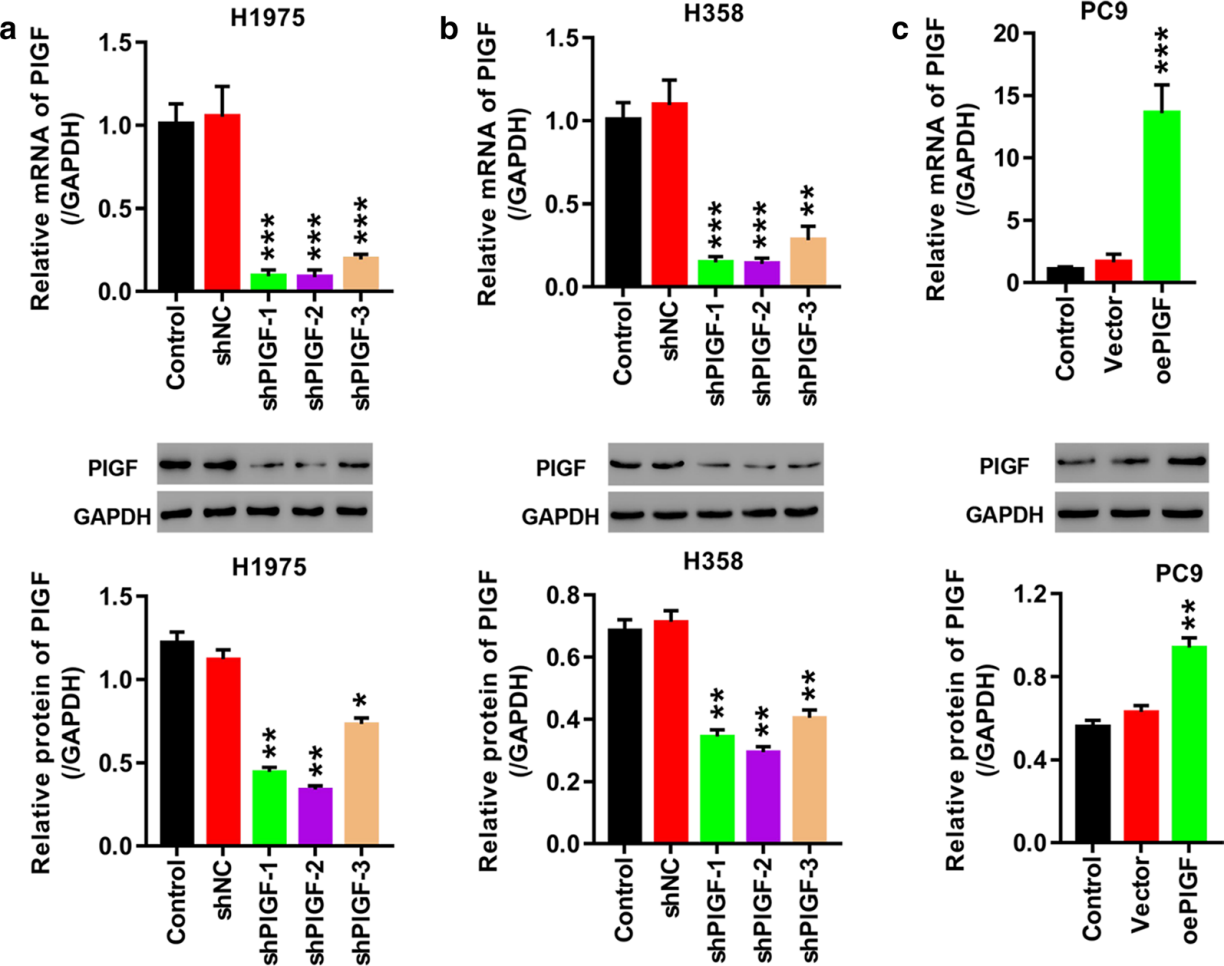
Knockdown and overexpression of PlGF by lentivirus infection. **a**, **b** H1975 (**a**) and H358 cells (**b**) are infected with shNC, shPlGF-1, shPlGF-2, or shPlGF-3 to knock down PlGF. **c** PlGF is overexpressed in PC9 cells transfected with oePlGF. mRNA and protein of PlGF are examined, respectively. *p < 0.05, **p < 0.01, ***p < 0.001 *vs.* shNC or Vector

The expression of PlGF in H358 and H1975 cells was knocked down by lentivirus-mediated RNA interference. As shown in Fig. 2a, b, all the three shRNAs effectively repressed PlGF expression at mRNA level in H358 and H1975 cells (*p *< 0.01 *vs.* shNC), each with knockdown efficiency higher than 70%. At protein level the silencing efficiency of shPlGF-1, shPlGF-2 and shPlGF-3 was higher than 60% in H358 cells (Fig. 2b, *p *< 0.01). Nevertheless, in H1975 cells, unlike shPlGF-1 and shPlGF-2 which decreased PlGF protein by more than 60% (*p *< 0.01), shPlGF-3 failed to knockdown 50% of PlGF protein (Fig. 2a). Taken together, we decided to use shPlGF-1and shPlGF-2 to infect lung cancer cells for silencing PlGF in further experiments.

PC9 cells were transfected with oePlGF plasmid for overexpressing PlGF or empty plasmid. Figure 2c showed a more than tenfold elevation of PlGF mRNA (*p *< 0.001) and a onefold elevation of PlGF protein (*p *< 0.01) in the oePlGF-transfected PC3 cells compared to the vector-transfected cells, indicating successful overexpression of PlGF in PC9 cells.

### Hypoxia treatment led to up-regulation of HIF-1α and PlGF in H358 and H1975 cells

**Fig. 3:**
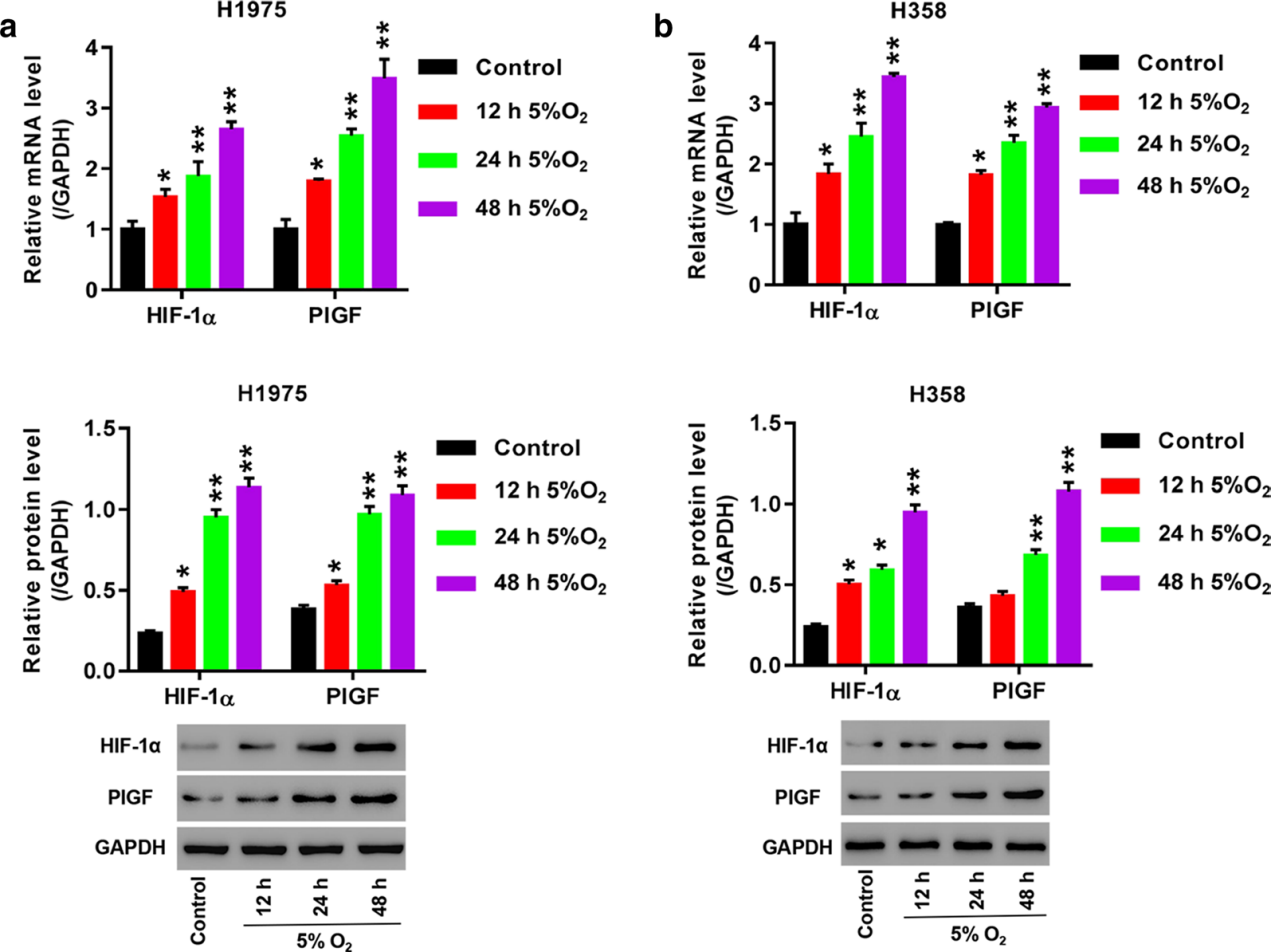
Hypoxia induced up-regulation of HIF-1α and PlGF in H1975 and H358 cells. **a**, **b** Cells are cultured under hypoxia (90% N_2_, 5% CO_2_ and 5% O_2_) or normoxia (5% CO_2_ and 95% air, control) for 12 h, 24 h, and 48 h. mRNA and protein levels of HIF-1α and PlGF in H1975 (**a**) and H358 (**b**) are compared between the hypoxic and normoxic conditions. *p < 0.05, **p < 0.01, ***p < 0.001 *vs.* Control

H358 and H1975 cells were cultured under hypoxia (5% O_2_ and 5% CO_2_) or normoxia (5% O_2_ and 95% air) for 48 h. mRNA and protein levels of HIF-1α and PlGF were examined, separately. Exposure of H358 and H1975 cells to hypoxia caused significantly increased mRNA levels of HIF-1α and PlGF at 12 h (*p *< 0.05), 24 h and 48 h (*p *< 0.01, Fig. 3a, b) compared to the cells exposed to normoxia. Similarly, in H1975 cells HIF-1α and PlGF protein were obviously elevated in response to hypoxia treatment for 12 h, 24 h and 48 h ((*p *< 0.05; *p *< 0.01; *p *< 0.01, Fig. 3a). In H358 cells hypoxia exposure for 24 h and 48 h (*p *< 0.05; *p *< 0.01) induced remarkable increases of HIF-1α and PlGF protein, yet, 12-h hypoxia exposure brought about significantly increased HIF-1α protein (*p *< 0.05) and insignificantly increased PlGF protein (*p *> 0.05, Fig. 3b). Therefore, we selected hypoxia treatment for 24 h in further experiments. Moreover, increases of mRNA and protein levels of HIF-1α and PlGF showed a time-dependent trend in H358 and H1975 cells upon hypoxia treatment.

### Knockdown of PlGF abrogated the impact of hypoxia on H358 and H1975 cells

**Fig. 4:**
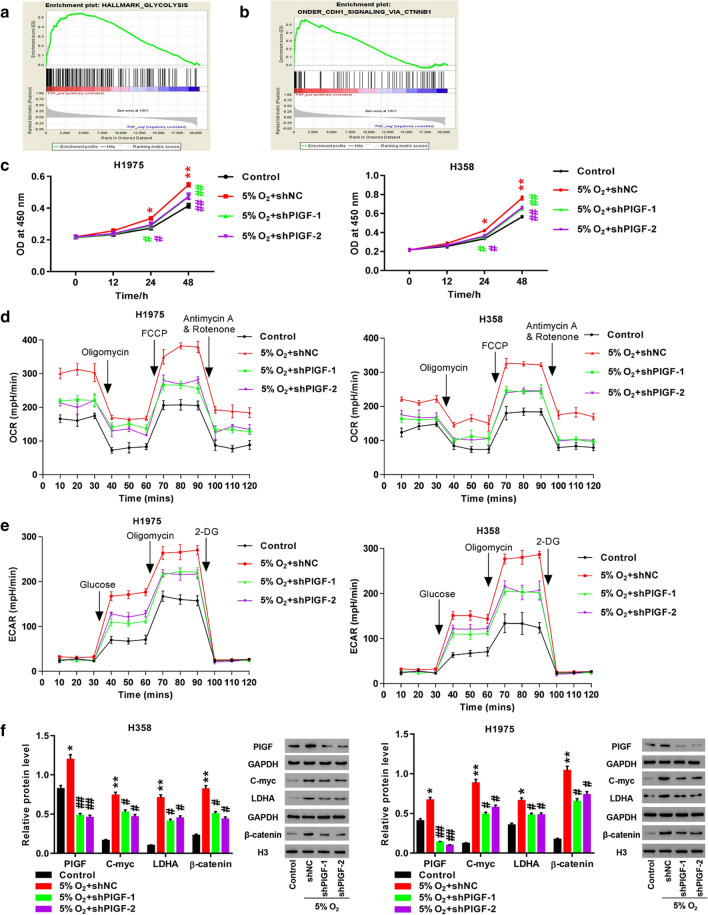
Knockdown of PlGF potently reversed hypoxia-induced proliferation and glycolysis. **a**, **b** Enrichment results of glycolysis (**a**) and β-catenin (**b**); **c** Measurement of cell proliferation by CCK-8 assay; **d** Evaluation of OCR level by cell mito stress test assay; **e** Evaluation of ECAR level by glycolysis stress test assay; **f** Detection of PlGF, C-myc, LDHA, and β-catenin by Western blot. H1975 and H358 cells are infected with shNC, shPlGF-1 or shPlGF-2, followed by exposure to hypoxic condition for 24 h. *p < 0.05, **p < 0.01 vs. Control; #*p *< 0.05, ##*p *< 0.01 *vs.* 5% O_2_ + shNC

We further investigated the effect of PlGF on hypoxia-induced cell proliferation, OCR and ECAR levels by knocking down PlGF in H358 and H1975 cells. Enrichment analysis revealed that PlGF up-regulation in LUAD was associated with glycolysis andβ-catenin pathway (Fig. 4a, b). CCK-8 assay was utilized for measurement of cell proliferation. We observed significant increases of OD values at 450 nm in shNC-infected H358 and H1975 cells exposed to hypoxia compared to the cells exposed to normoxia at 24 h and 48 h (*p *< 0.05; *p *< 0.01, Fig. 4c). Furthermore, OD values were significantly decreased in the shPlGF-1-or shPlGF-2-infected cells in comparison with the shNC-infected cells under hypoxia at 24 h and 48 h (*p *< 0.05; *p *< 0.01, Fig. 4c).

We performed cell mito stress test assay to evaluate mitochondrial respiration by measurement of OCR pattern. As shown in Fig. 4d, hypoxia and shNC infection resulted in observable increases of OCR levels in H358 and H1975 cells after subsequent exposure to oligomycin, FCCP, and rotenone and antimycin A. Infection ofH358 and H1975 cells with shPlGF-1 or shPlGF-2 partly ameliorated the increases of OCR levels induced by hypoxia.

Glycolysis stress test assay was performed to assess glycolytic respiration by measurement of ECAR. When using the assay, cells were cultured in the medium sequentially injected with glucose, oligomycin and 2-DG. In H358 and H1975 cells ECAR levels were remarkably elevated in response to hypoxia treatment after injection of glucose and oligomycin, and before 2-DG treatment (Fig. 4e). The hypoxia-induced elevations of ECAR levels were partly reversed inH358 and H1975 cells by shPlGF-1 or shPlGF-2 infection (Fig. 4e).

The above results suggest that hypoxia promoted cell proliferation and increased OCR and ECAR levels in H358 and H1975 cells, which could be partly alleviated by PlGF knockdown. It reports that hypoxia augments lung cancer development through activating Wnt signaling [32]. Activation of Wnt/β-catenin pathway advances tumor progression by promoting transcription of C-myc [33] which directly regulates LDHA [18]. In order to decipher the underlying molecular mechanisms of PlGF in cell proliferation and glycolysis, we further examined protein expression of PlGF, β-catenin, C-myc and LDHA in H358 and H1975 cells by using Western blot. Not unexpectedly, hypoxia exposure resulted in significant up-regulation of PlGF (*p *< 0.05), C-myc (*p *< 0.01), LDHA (*p *< 0.05), and β-catenin (*p *< 0.01) in H358 and H1975 cells at protein level (Fig. 4f). Moreover, PlGF knockdown by shPlGF-1 or shPlGF-2 infection significantly compromised the stimulation of hypoxia (Fig. 4f). These results revealed that hypoxia up-regulated PlGF, β-catenin, C-myc and LDHA in H358 and H1975 cells, which could be partly mitigated by PlGF knockdown.

### Suppression of Wnt/β-catenin pathway abolished the enhancement of cell proliferation and glycolysis caused by PlGF overexpression in PC9 cells

**Fig. 5:**
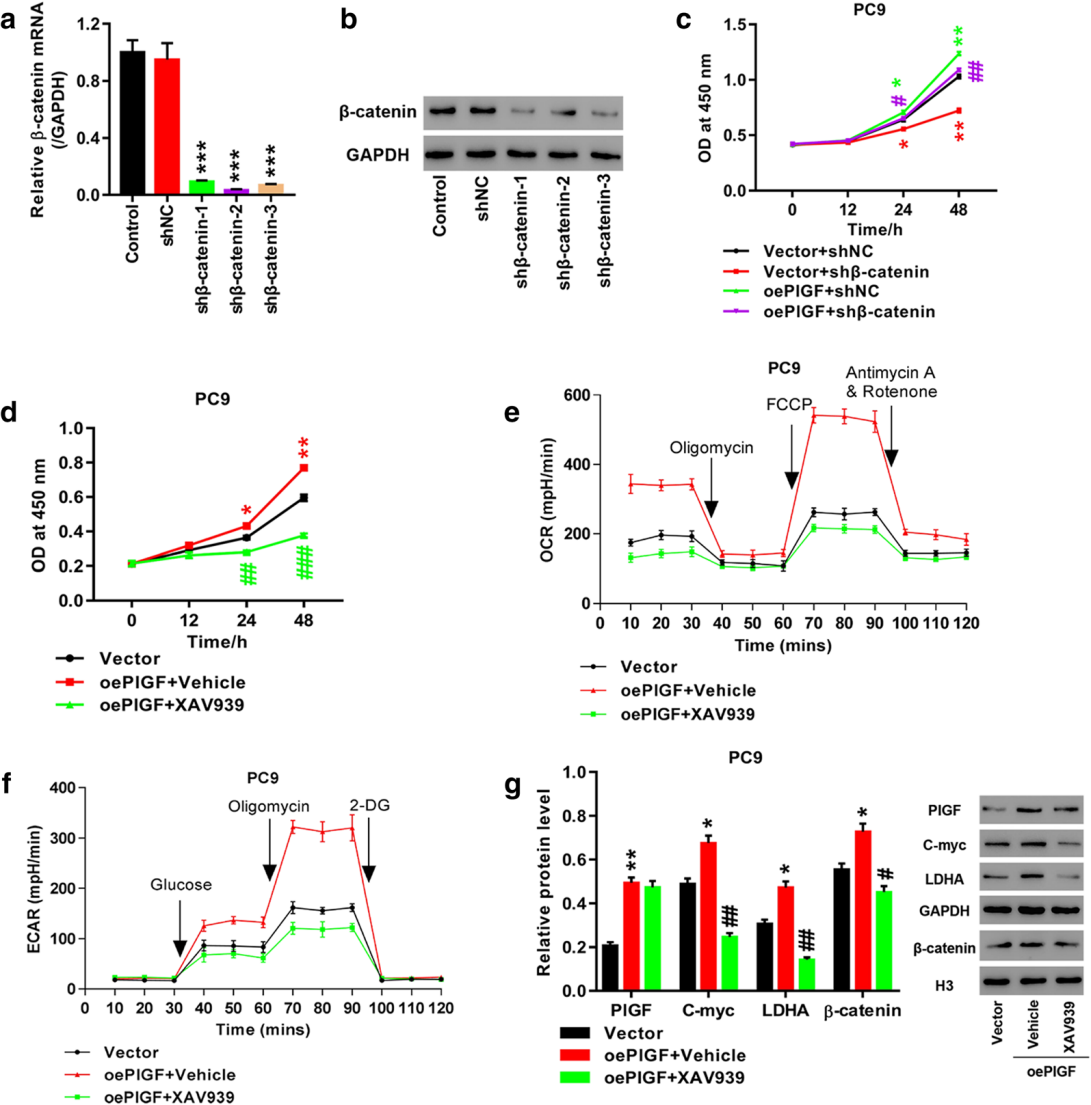
Wnt/β-catenin pathway inhibition eliminated the promoting effect of PlGF knockdown on proliferation and glycolysis of PC9 cells. **a**, **b** The efficiency of β-catenin knockdown in PC9 cells were detected by Q-PCR and western blot. **c** After treatment of PlGF overexpression combined with β-catenin knockdown, cell proliferation was measured by CCK-8 assay; PC9 cells are transfected with oePlGF or vector, and then are treated with XAV939, specific inhibitor of Wnt/β-catenin pathway, for 24 h. **d** Measurement of cell proliferation byCCK-8 assay; **e** Evaluation of OCR level by cell mito stress test assay; **f** Evaluation of ECAR level by glycolysis stress test assay; **g** Detection of PlGF, C-myc, LDHA, and β-catenin by Western blot. *p < 0.05, **p < 0.01 *vs.* Vector; #*p *< 0.05, ##*p *< 0.01, ###*p *< 0.001 *vs*. oePlGF + Vehicle. oePlGF represents PlGF overexpression. shβ-catenin represents β-catenin knockdown

In order to further determine whether Wnt/β-catenin pathway participated in mediating the impact of PlGF on lung cancer cells, we overexpressed PlGF in PC9 cells by transfection with oePlGF plasmid, which were then knocked down β-catenin (Fig. 5a, b) or incubated with XAV939 (10 μmol/L), inhibitor of Wnt/β-catenin pathway, for 24 h. As shown in Fig. 5c, PlGF overexpression significantly stimulated cell proliferation, which was inhibited by β-catenin knockdown. Consistently, Incubation with XAV939 markedly reversed PlGF overexpression-induced cell proliferation (*p *< 0.05, Fig. 5d), OCR (Fig. 5e) and ECAR (Fig. 5f) levels, and the expression of β-catenin, C-myc and LDHA protein (*p *< 0.05, Fig. 5g). These observations suggest that like hypoxia exposure, PlGF overexpression exerted a stimulatory effect on cell proliferation, glycolysis, protein expression of C-myc and LDHA, which was mediated via Wnt/β-catenin pathway.

### PlGF knockdown induced a suppression of tumor growth in nude mice

**Fig. 6:**
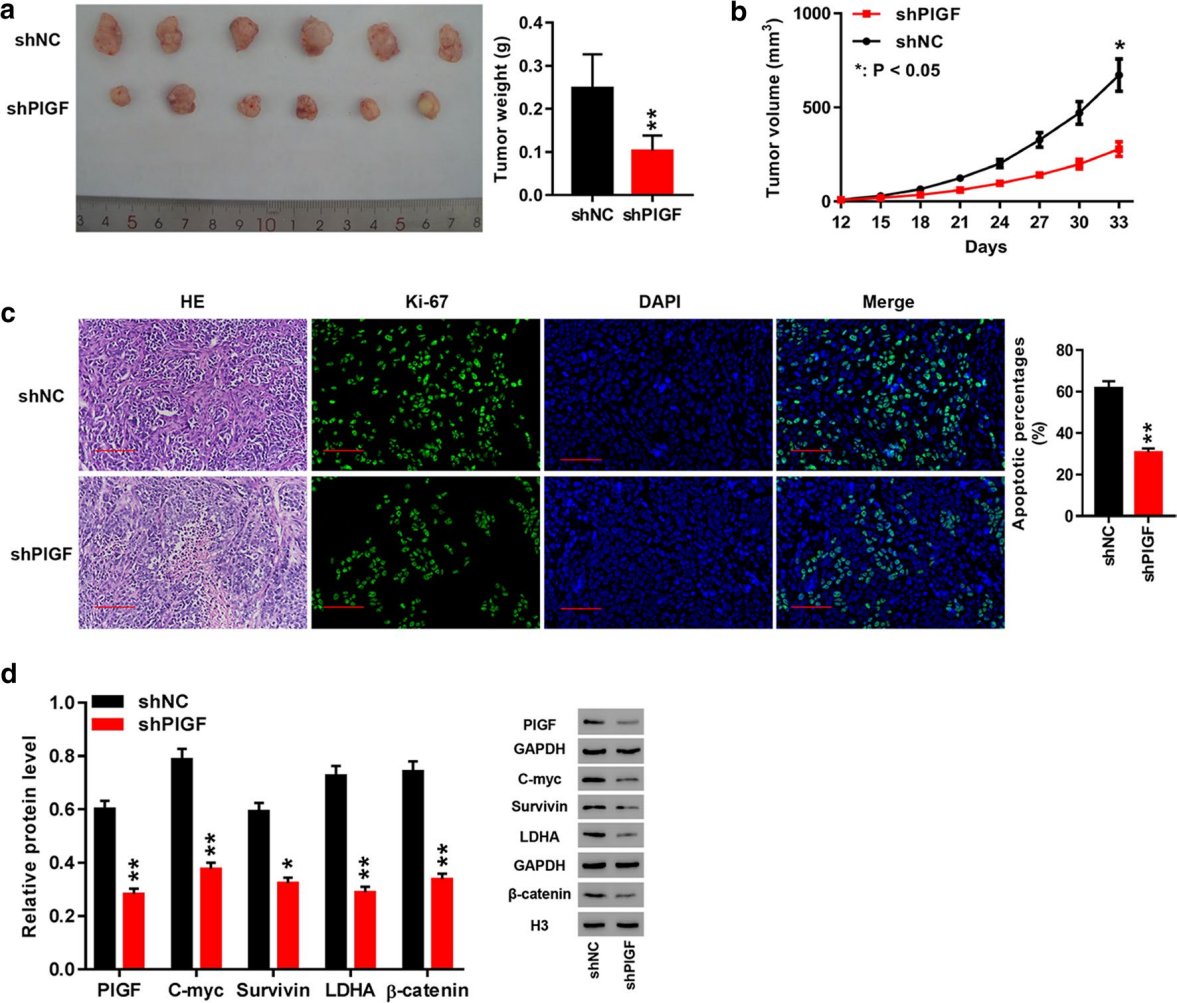
PlGF knockdown significantly repressed tumor growth in nude mice. **a** Final weight of shNC and shPlGF xenograftes in mice; **b** Tumor growth curve of shNC and shPlGF xenografts in mice; **c** HE staining (200 × magnification) and Ki-67 immunofluorescence (400 × magnification) in shNC and shPlGF xenografts; **d** Western blot analysis of PlGF, C-myc, survivin, LDHA, and β-catenin in shNC and shPlGF xenografts. *p < 0.05, **p < 0.01 *vs.* shNC

We further analyzed the biological activity of PlGF in tumor xenografts by injecting H1975 cells infected with shPlGF or shNC into nude mice. The results showed that the shPlGF xenografts had lighter tumor weight (*p *< 0.01), smaller tumor volume (*p *< 0.05) and weaker positive staining for Ki-67, a biomarker of cell proliferation [34], compared to the shNC xenografts (Fig. 6a–c). Western blot analysis of PlGF, β-catenin, C-myc and LDHA in tumor tissues revealed that depletion of PlGF caused significant reductions of protein levels of C-myc, β-catenin, and LDHA in shPlGF xenografts, which were in accordance with the results in H358 and H1975 cells (Fig. 6d). It implied that PlGF was involved in modulating tumor growth by modulating protein expression of β-catenin, C-myc, and LDHA.

## Discussion

PlGF has been found to have a multifaceted role in cancer progression, angiogenesis and prognosis [35]. PlGF participates in regulating invasion of several types of cancers, such as ovarian cancer [36, 37] and cutaneous T cell lymphoma [38]. Moreover, there is evidence that PlGF expression in tumor tissue could be a promising biomarker of therapeutic efficacy of ramucirumab in patients with gastric cancer [39]. Based on TCGA data, bioinformatics analysis of the present study revealed that PlGF was up-regulated in tumor tissue and two LUAD cell lines (H358 and H1975). Moreover, in vivo data obtained in the current study suggested that PlGF silencing suppressed tumor growth as seen by lighter and smaller tumors with decreased Ki-67 expression after PlGF knockdown in nude mice. These results supported the significance of PlGF in promoting LUAD progression, which is in concordance with previous studies [21–23].

PlGF has been reported to be overexpressed as a result of up-regulation of HIF induced by hypoxia [40]. Similarly, in our study western blot and RT-PCR results consistently showed that PlGF and HIF-1α were markedly up-regulated in H358 and H1975 cells on exposure to hypoxia. Hypoxia is closely associated with angiogenesis, apoptosis, and treatment resistance of lung cancer, and has emerged as a promising target for therapies [41]. In our study, along with significantly increased OD values that were observed in H358 and H1975 cells after hypoxia exposure, we observed remarkable increases of OCR and ECAR levels, demonstrating that hypoxia stimulated cell proliferation and glycolysis in lung cancer. Moreover, PlGF knockdown successfully attenuated the stimulation of cell proliferation and glycolysis of lung cancer cells by hypoxia.

LDHA is a cytosolic enzyme primarily implicated in converting pyruvate into lactate in anaerobic glycolysis [42]. Up-regulation of LDHA has been demonstrated in several cancers and associates with carcinogenesis and tumor progression [43]. Results obtained from the current study showed that hypoxia exposure brought about elevations of expression of C-myc and LDHA in LUAD cells, which were alleviated by knocking down PlGF. Moreover, our study provided in vivo evidence that PlGF silencing caused down-regulation of C-myc and LDHA by examining tumor xenografts harvested from mice and in vitro evidence that PlGF overexpression resulted in up-regulation of C-myc and LDHA. These results further confirmed that the stimulatory effect of hypoxia on glycolysis could be successfully reversed by PlGF knockdown in LUAD. In light of above findings, we speculate that PlGF may be a promising therapeutic target for LUAD.

Wnt/β-catenin pathway has a profound role in proliferation, apoptosis of lung cancer cells, affecting tumorigenesis and prognosis [44, 45]. Targeting Wnt/β-catenin raises the possibility of developing novel drugs against lung cancer [46]. Hypoxia activates Wnt/β-catenin pathway in non-small cell lung cancer and hepatocellular carcinoma [47, 48]. In the current study, hypoxia exposure and PlGF overexpression had similar stimulatory effect on expression of β-catenin in lung cancer cells. Moreover, PlGF silencing obviously reduced β-catenin expression in lung cancer cells and tumors xenografts. These findings imply that Wnt/β-catenin pathway may be a downstream target of PlGF in LUAD, which is in agreement with a report supporting involvement of PlGF in apoptosis of gastric cancer stem cells through Wnt signaling pathway [49]. Furthermore, our study revealed that inhibition of Wnt/β-catenin pathway effectively eliminated the stimulatory effect of PlGF overexpression on cell proliferation and expression of C-myc and LDHA. It implied that PlGF might modulate cell proliferation and glycolysis of LUAD via downstream Wnt/β-catenin pathway.

This study offers novel insights into the molecular mechanisms of PlGF in LUAD. Yet, PlGF is not overexpressed and Wnt/β-catenin pathway is not inhibited in mice models to validate the associations of PlGF and Wnt/β-catenin pathway. Further experiments are necessary to address this issue.

## Conclusion

Taken together, this study suggests that PlGF knockdown ameliorates the stimulation of cell proliferation and glycolysis caused by hypoxia by suppressing Wnt/β-catenin pathway. This study presents PlGF as a potential therapeutic target for LUAD and aids in dissecting the detailed mechanisms underlying the biological functions of PlGF in LUAD.

## Data Availability

All the original data of the current study are available from the corresponding author on reasonable request.

## Abbreviations

PlGF: Angiogenic placental growth factor
LUAD: Lung adenocarcinoma
LDHA: Lactate dehydrogenase A
HIF-1: Hypoxia-inducible factor-1
VEGF: Vascular endothelial growth factor
OCR: Oxygen consumption rates
ECAR: Extracellular acidification rate
TCGA: The Cancer Genome Atlas
IHC: Immunohistochemistry
16HBE: Human bronchial epithelial cell
qRT-PCR: Quantitative real-time PCR
CCK-8: Cell counting kit-8
OD: Optimal density
2-DG: 2-deoxyglucose
IF: Immunofluorescence
HE: Hematoxylin-eosin
ANOVA: One-way analysis of variance
